# Supplementary figures and images for: High Tumoral STMN1 Expression Is Associated with Malignant Potential and Poor Prognosis in Patients with Neuroblastoma
Source: Cancers (Basel). 2023 Sep 8;15(18):4482. doi: 10.3390/cancers15184482 (PMC10526320; doi:10.3390/cancers15184482)

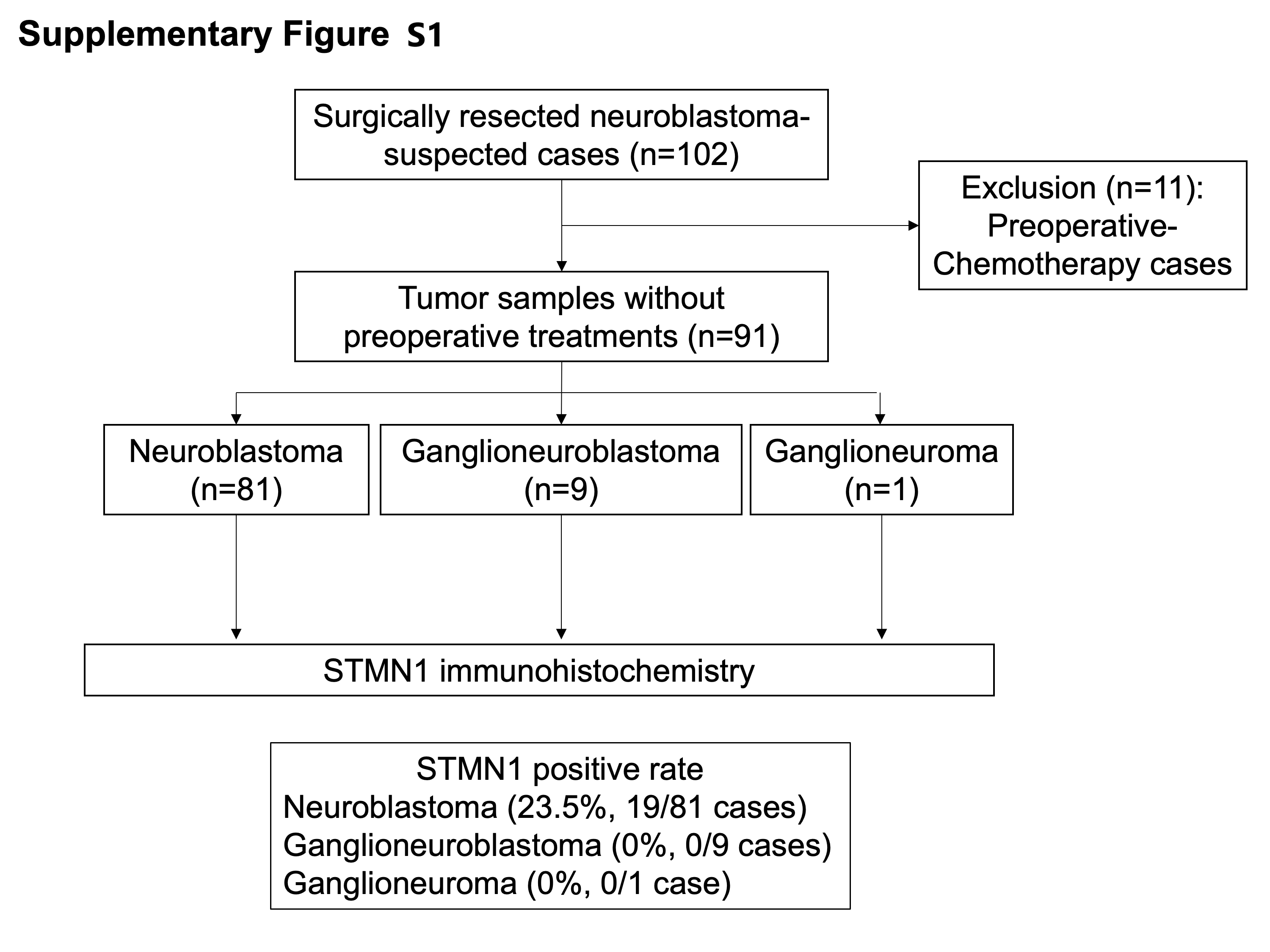

Supplement: Supplementary file 1 [file cancers-15-04482-s001.zip › cancers-2508144-supplementary/Supplementary Figure S1.tiff]

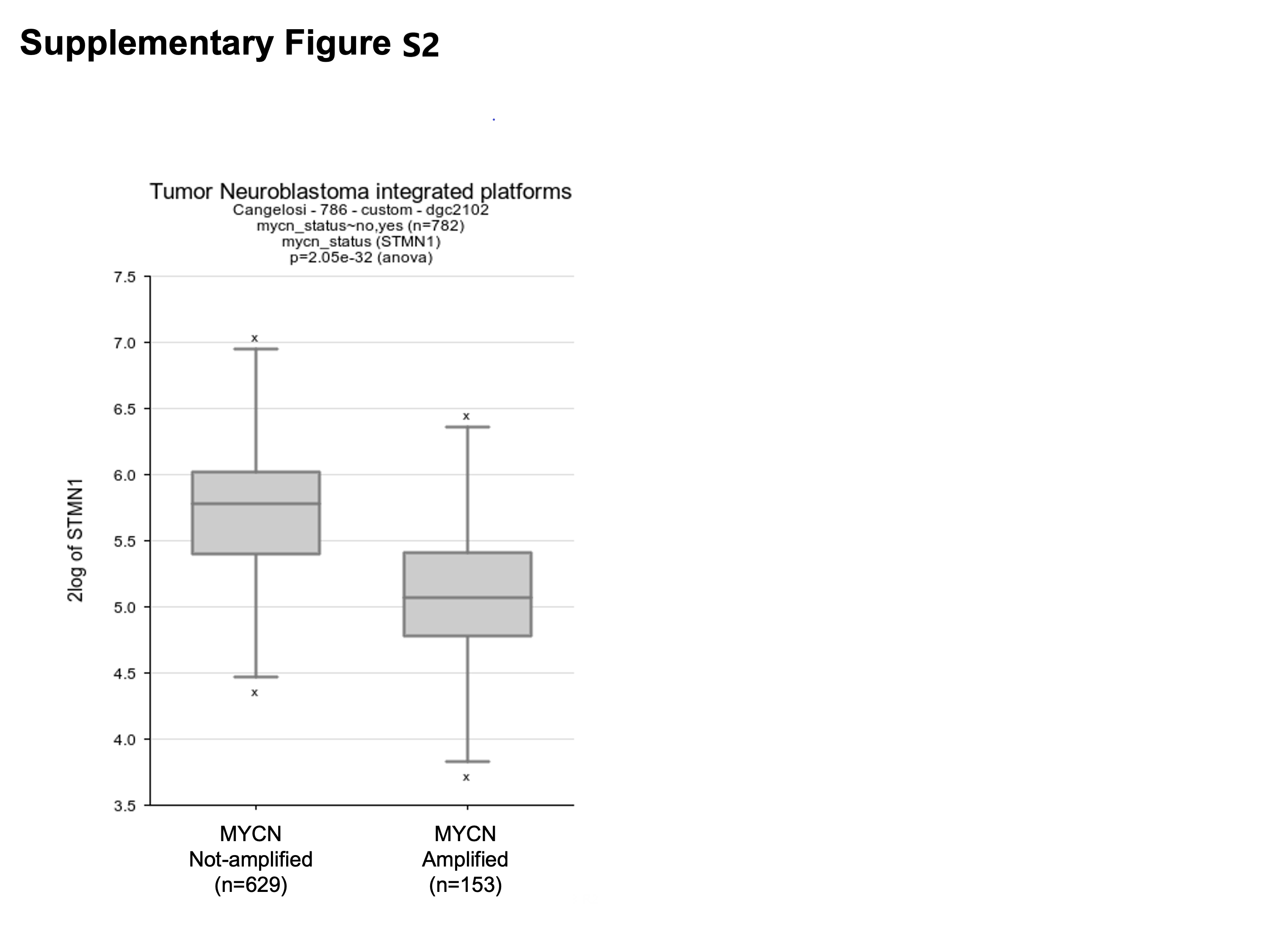

Supplement: Supplementary file 1 [file cancers-15-04482-s001.zip › cancers-2508144-supplementary/Supplementary Figure S2.tiff]
